# Effect of isokinetic muscle strength training on knee muscle strength, proprioception, and balance ability in athletes with anterior cruciate ligament reconstruction: a randomised control trial

**DOI:** 10.3389/fphys.2023.1237497

**Published:** 2023-09-19

**Authors:** Kun Wang, Liang Cheng, Bingcheng Wang, Benxiang He

**Affiliations:** ^1^ Post-Doctoral Scientific Research Workstation of Affiliated Sport Hospital, Chengdu Sport University, Chengdu, China; ^2^ School of Sports Medicine and Health, Chengdu Sport University, Chengdu, China; ^3^ Jinling Hospital, General Hospital of Eastern Theater Command, Nanjing, China; ^4^ Sichuan Academy of Traditional Chinese Medicine, Chengdu, China

**Keywords:** elite athletes, anterior cruciate ligament, equivalent speed training, postural control ability, muscle strength

## Abstract

**Objective:** This study aimed to investigate the effects of regular isokinetic muscle strength training on knee muscle strength, proprioception, and balance ability in athletes after anterior cruciate ligament (ACL) reconstruction.

**Methods:** Forty-one athletes who underwent ACL reconstruction were randomly divided into the experimental (*n* = 21) and control (*n* = 20) groups. The experimental group used an isokinetic muscle strength tester for 4 weeks (five times/ week) of knee flexion and extension isokinetic muscle strength training. The control group used the knee joint trainer (pneumatic resistance) for the same exercise regimen as the experimental group.

**Results:** 1) Four weeks when compared with the baseline. Experimental group: the knee flexion and extension PT (60°/s and 240°/s) increased by 31.7%, 40.3%, 23.4%, and 42.9% (*p* < 0.01), and the flexion muscular endurance increased by 21.4% and 19.7% (*p* < 0.01). The flexion and extension kinaesthesia and the 30° and 60° position sense decreased by 36.2%, 32.3%, 40.0%, and 18.9% (*p* < 0.05). The anterior–posterior and medial–lateral displacement and speed decreased by 30.2%, 44.2%, 38.4%, and 24.0% (*p* < 0.05). Control group: the knee peak torque (60°/s) increased by 18.8% (*p* < 0.01). The anterior–posterior and medial–lateral displacement and speed decreased by 14.9%, 40.0%, 26.8%, and 19.5% (*p* < 0.01). 2) After 4 weeks, compared with the control group, the knee flexion and extension peak torque (60°/s), extension, peak torque (240°/s), and extension muscular endurance of the treatment group increased to varying degrees (*p* < 0.05). However, the kinaesthesia, 30° position sense, and anterior–posterior displacement decreased to varying degrees (*p* < 0.05).

**Conclusion:** Adding regular isokinetic muscle strength training to rehabilitation training further improved the knee flexion and extensor strength and extensor endurance of athletes with ACL reconstruction, as well as enhanced the kinaesthesia and 30° position sense and the balance between the anterior and posterior directions. However, the treatment had limited effects on knee flexion kinaesthesia and muscle endurance.

## Introduction

Athletes increase their risk of knee injury during starting acceleration, emergency stop and deceleration, rotation, or ground kick practices (Barber-Westin and Noyes, 2016; Bram et al., 2021). Anterior cruciate ligament (ACL) fracture is a common knee injury in athletes (Larwa et al., 2021; Kotsifaki et al., 2023). It causes tibial advancement and internal rotation, which cause knee instability and further damage to the meniscus and articular surface (Kittl et al., 2016; Sonnery-Cottet et al., 2017). ACL fracture leads to the destruction of the mechanical structure of the knee (Blaker et al., 2021; Tayfur et al., 2021). Knee-joint static stability can be restored by bone–patellar tendon–bone reconstruction, but the decline in postoperative joint muscle strength can cause dynamic knee joint instability (Brophy et al., 2022; Takagi et al., 2022). Restoring the stability and mechanical structure of the knee is the main purpose of ACL reconstruction (Lin et al., 2020). Previous studies have shown that the proportion of ACL injuries in female athletes is 2.1–2.4 times higher than that in male athletes (Kaeding et al., 2017). Female athletes still exhibit loss of knee extensor strength 2 years after ACL reconstruction (Buckthorpe et al., 2019). A single-centre database includes 1,362 athletes with ACL reconstruction, and approximately 83.7% athletes returned to sports within 2 years of reconstruction (Toale et al., 2021). Therefore, the motor function of the knee joint must be restored early after surgery.

ACL is rich in proprioception (muscles, tendons, and joints generated during movement or rest, which play an important role in maintaining joint stability) (Cheng et al., 2017). The afferent device can provide static mechanical stability for the knee joint and maintain the stability of the knee joint with the help of nerve reflex (Lee et al., 2009). The absence of proprioception after ACL reconstruction in athletes is detrimental to the recovery of motor function, and the recovery of proprioception delays the improvement in muscle strength (Laboute et al., 2019; Ghaderi et al., 2020). In addition, ACL reconstruction in athletes is accompanied with a decline in balance ability (Güzel et al., 2022; Rahova et al., 2022), and a good balance is important to return to competitions.

Isokinetic muscle strength training produces maximum output at any angle within the joint range of motion; it is safer and more effective than conventional muscle strength training (e.g., using fixed resistance) (Cheng et al., 2019; Cheng and Jiang, 2020). Isokinetic muscle strength training is widely used in the rehabilitation training of athletes (Vidmar et al., 2020a; Saral et al., 2022). Thus, ACL reconstruction with isokinetic training of athletes has also been reported. Previous studies have shown that isokinetic muscle strength training promotes the recovery of knee flexor and extensor muscle strength (60°/s) in athletes with ACL reconstruction (Zhang et al., 2016). Previous studies found that rehabilitation with additional isokinetic training improves muscle strength at 4 weeks after ACL reconstruction in adult athletes (Tsaklis and Abatzides, 2002). Two weeks of isokinetic muscle strength training for the ACL reconstruction in young athletes can improve the quality and strength of quadriceps (Vidmar et al., 2020b). Unfortunately, these studies did not report changes in the knee explosive force (e.g., knee tested at 240°/s) and muscle endurance, and changes in proprioception and balance ability were not analysed. Good explosive power and muscular endurance, proprioception, and balance ability in athletes are important to maintain a high level of competitive ability (Gidu et al., 2022).

To clarify the effects of isokinetic muscle strength training in athletes after ACL reconstruction, we subjected athletes with ACL reconstruction to regular isokinetic muscle strength training, analysed changes in human posture control ability (joint muscle strength, proprioception, and balance), and determined the influence of scientific rehabilitation training to restore the competitive ability in athletes so that they may return to competitions as soon as possible. Study hypothesis: We hypothesised that adding isokinetic muscle strength training to rehabilitation training can improve the knee muscle strength, proprioception, and balance ability of athletes with ACL reconstruction.

## Materials and methods

### Participants

This study was approved by the Ethics Committee of Chengdu Sport University (No: 201702). From June 2017 to December 2022, athletes with ACL reconstruction from Sichuan Province, China, were recruited. The inclusion criteria were as follows: unilateral autologous bone–patellar tendon–bone reconstruction; national athlete (sports performance: individual or team events, ranked top 8 in China, i.e., domestic championship); and early full-angle knee extension, early weight bearing, and progressive closed chain training. The study was compliant with the Declaration of Helsinki, and signed informed consent was obtained. The exclusion criteria were as follows: meniscectomy, fracture, previous history of surgery on other parts of the knee, and other forms of muscle or bone injury in the lower limb.

During this period, 42 subjects were recruited (judo: male/female, 3/5; wrestling: male/female, 2/4; kung fu: male/female: 1/2; football: male/female, 3/6; volleyball: male/female, 1/3; basketball: male/female, 1/2; weightlifting: male/female, 1/0; hockey: male/female, 1/3; tennis: male/female, 1/2; and taekwondo: male/female, 0/1). They were divided into the experimental group (*n* = 21, male/female: 7/14) and the control group (*n* = 21, male/female: 7/14). Throughout the 4-week experimental intervention, one sample in the control group dropped out for personal reasons. Finally, 21 cases in the experimental group and 20 cases in the control group completed the whole experimental process (Table 1). The differences in the mean values of age, height, weight, and years of training between the experimental and control groups were not statistically significant (*p* > 0.05).

**TABLE 1:** Basic information and rehabilitation training and treatment.

Essential information	Experimental group (*n* = 21)	Control group (*n* = 20)
Age (y)	21.6 ± 3.2	22.2 ± 2.8
Height (cm)	176.0 ± 8.2	175.6 ± 7.6
Body mass (kg)	69.5 ± 11.3	70.0 ± 12.5
Years of training period (y)	10.3 ± 2.6	9.7 ± 3.1
Treatment (weeks 1–2)	Ultrasound therapy, half-squat practice, gait training, MRS and other long-range training and proprioception practice, KAT balance training, and ice compress	
Treatment (weeks 3–4)	On the basis of the week 1–2 treatment, KAT dynamic balance exercises and weight loss running–walking training were added	
Treatment (weeks 5–6)	On the basis of the treatment in weeks 3–4	
Treatment (weeks 7–8)	On the basis of the treatment in weeks 5–6, special movement training such as balance plate and cardiopulmonary function should be added	

First, all subjects underwent the same intensity of rehabilitation training with the same dose of ultrasound and other treatments (Table 1). After 4 weeks of ACL reconstruction, the experimental group was subjected to isometric knee flexion and extension muscle training for 4 weeks using a German IsoMed-2000 isokinetic muscle tester. The control group was trained with the same exercise dose of knee flexion and extension using the Finnish HUR knee pneumatic trainer as the experimental group (50%–70% RM resistance was used for training).

### Isokinetic muscle strength training and joint muscle strength tests

The IsoMed-2000 isokinetic testing apparatus (IsoMed-2000 dynamometer; D & R Ferstl GmbH, Hemau, Germany) was used to perform the isokinetic training of flexion and extension at 60°/s and 240°/s (five sets of 12 repetitions/set with 1 min rest between sets and 5 min between angles) for 4 weeks (five repetitions/week) on the reconstructed knee of the ACL side (80° joint mobility) of the subjects in the experimental group. All subjects were tested before and after 4 weeks of ACL reconstruction (60°/s, five repetitions; 240°/s, 25 repetitions). In the 240°/s test, the ratio of the 21st to 25th total work (knee flexion or extension) to the 1st to 5th total work (knee flexion or extension) was measured. This ratio was used to reflect the muscle endurance of the subject’s knee joint in the flexion or extensor muscle group. The closer this ratio is to 1, the better the endurance level of the knee flexion or extensor muscle group in the subjects (Cheng et al., 2019; Cheng and Jiang, 2020).

### Proprioception test

The proprioceptive tests included kinaesthetic and positional tests (Cheng et al., 2017). For kinaesthetic tests, a custom-made knee kinaesthetic device with a reliability of 0.93 for the knee test was used with good repeatability and reliability (Cheng et al., 2017). The device was equipped with a pedal that could be rotated around a single axis at an angular velocity of 0.3°/s. During the test, the subject was in a seated position with the dominant leg flat on the pedal, keeping the hip, knee, and ankle at 90°. By increasing the weight of the counterbalance, its weight could be matched with the weight of the leg (dominant leg) being tested in the subjects. This step helped compensate for the gravity of the dominant leg (eliminating the weight of the subject’s leg). Subjects wore headphones and eye protection (to exclude external sound and visual influences). The pedal randomly rotated in a certain direction (up or down) to flex or extend the knee joint; as soon as the subject perceived the movement of the foot and the direction of movement, he/she pressed the hand-held button to stop the rotating pedal. The smaller the angle of rotation of the pedal, the better the kinaesthetic perception. In the position perception test, we used an IsoMed-2000 isokinetic testing apparatus (IsoMed-2000 dynamometer; D & R Ferstl GmbH, Hemau, Germany) (Liu et al., 2019). The subject wore headphones and an eye mask (to exclude external sound and visual influences) and maintained the knee joint in the testing position and allowed the isometric instrument power head (angular velocity of 1°/s) to automatically flex and extend to the pre-set angles of 30° and 60°. When the angle returned to 0, the subject was allowed to move to the specified position (after the button movement automatically stopped). The difference between the actual angle and the pre-set angle was recorded, and each angle test was performed three times to determine the average value. The smaller the angle, the better the proprioception.

### Balance ability test

On the ACL reconstruction side, one foot (bare foot) stood on a 3D measuring bench (Kistler, Switzerland) for 10 s; this step was repeated three times (1 interval = 1 min) (Rogind et al., 2003). The test indicators were the maximum displacement, average speed of the pressure centre in the anterior–posterior (AP) direction, and average speed of the pressure centre in the medial–lateral (ML) direction. Large values indicated poor balance ability.

### Statistical analysis

Using SPSS 20, the data were treated with mean value ± standard deviation. The 2 (group) × 2 (time) design was considered (Cheng et al., 2021). The normality of the data was first measured using the Shapiro–Wilk test, using two-way ANOVA for group and time interaction. If an interaction was observed, we then determined whether a separate effect of time or group exists. Otherwise, we determined whether a main effect exists. Multiple comparisons were performed using the Bonferroni adjustment (significance level of *α* = 0.05).

## Results

**FIGURE 1:**
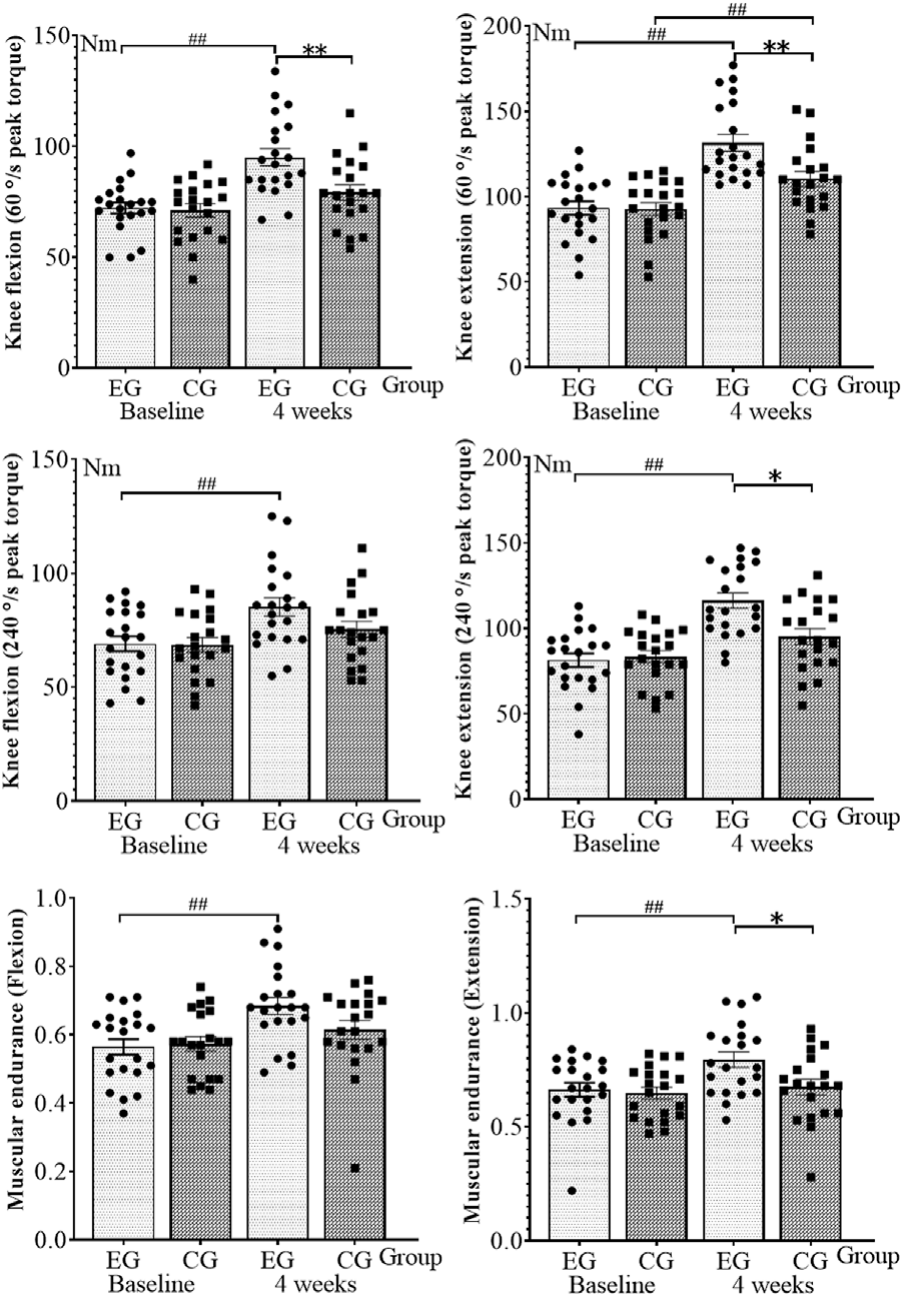
Knee muscle strength test results. EG, experimental group; CG, control group. *p < 0.05 and **p < 0.01 (after 4 weeks of intervention, between-group comparisons at the baseline and 4 weeks); ^##^ *p* < 0.01 (within-group comparisons at the baseline and 4 weeks).

**FIGURE 2:**
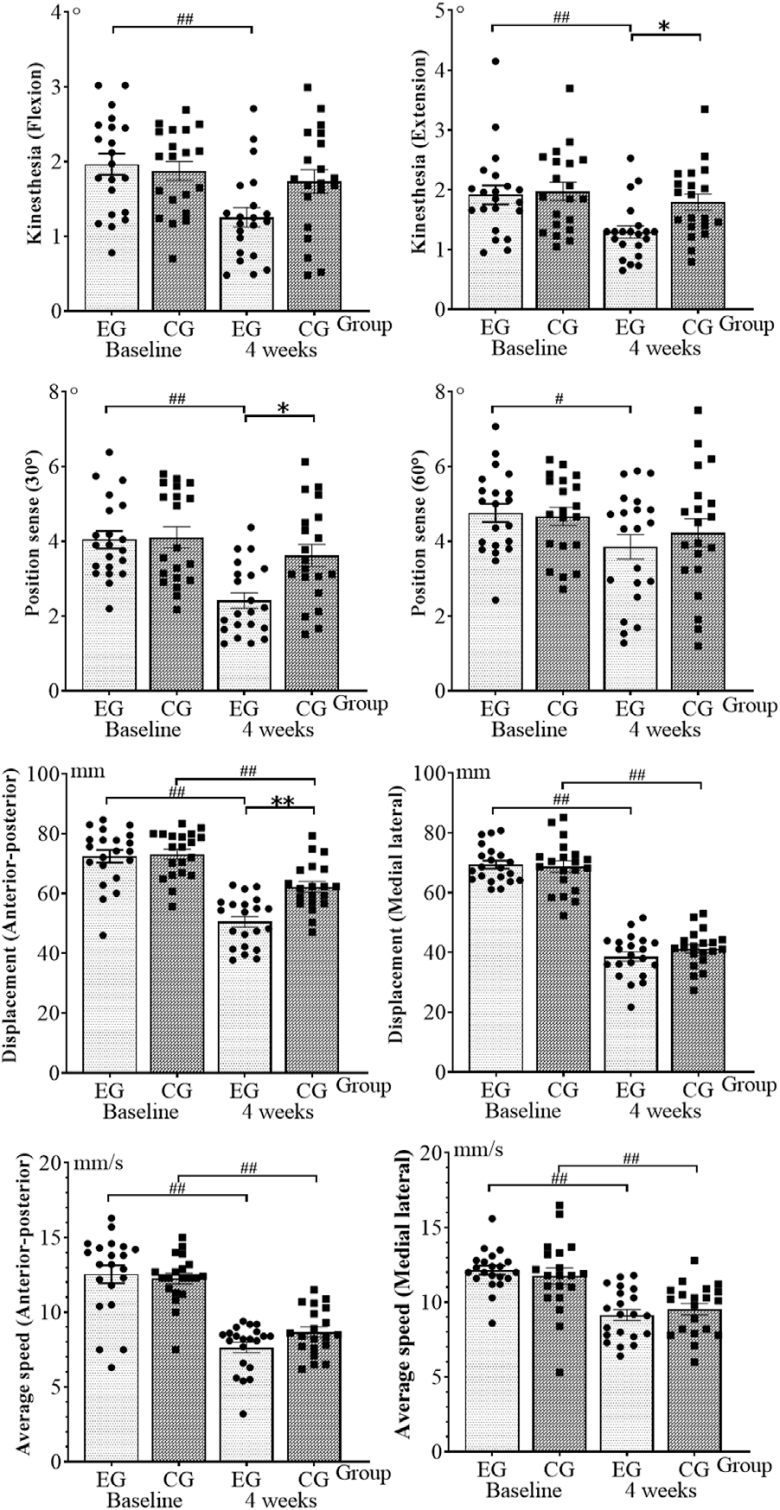
Results of proprioceptive and balance ability tests. EG, experimental group; CG, control group. *p < 0.05 and **p < 0.01 (after 4 weeks of intervention, between-group comparisons at the baseline and 4 weeks); ^#^ *p* < 0.05 and ^##^ *p* < 0.01 (within-group comparisons at the baseline and 4 weeks).

With an additional 4 weeks of isokinetic muscle strength training to rehabilitation training, the knee muscle strength test results of athletes with ACL reconstruction are shown in Figure 1. The proprioception and balance ability results are shown in Figure 2. The normality of the data was first measured using the Shapiro–Wilk test, showing that all the measurements exhibited normal distribution. No significant difference was observed between the baseline experimental and control data (*p* > 0.05).

In two-way ANOVA, group and time interactions were observed for knee flexion (F = 5.149, *p* = 0.026) and extension (F = 5.554, *p* = 0.021) PT (60°/s), extension (F = 7.728, *p* = 0.007) PT (240°/s), knee flexion kinaesthesia (F = 4.157, *p* = 0.045), 30° position sense (F = 5.085, *p* = 0.027), and AP displacement (F = 8.637, *p* = 0.004). Thus, we further determined whether a separate effect of group or time exists. Knee flexion (F = 1.767, *p* = 0.188) with PT (240°/s), knee flexion (F = 2.642, *p* = 0.108), and extension (F = 2.803, *p* = 0.098) interacted with muscular endurance. By contrast, extension kinaesthesia (F = 2.507, *p* = 0.117), 60° position sense (F = 0.583, *p* = 0.447), ML displacement (F = 1.046, *p* = 0.309), AP (F = 2.321, *p* = 0.132), and ML (F = 0.930, *p* = 0.338) interacted with mean speed. No interaction between group and time was observed.

At 4 weeks, compared with the baseline, the knee flexion (*p* < 0.001, η^2^ = 0.384) and extension (*p* < 0.001, η^2^ = 0.475) with PT (60°/s), knee flexion (*p* = 0.004, η^2^ = 0.194) and extension (*p* < 0.001, η^2^ = 0.462) with PT (240°/s), flexion (*p* = 0.001, η^2^ = 0.239), and extension muscular endurance (*p* = 0.006, η^2^ = 0.174) in the experimental group increased by 31.7%, 40.3%, 23.4%, 42.9%, 21.4%, and 19.7%, respectively, showing a statistically significant difference. The knee flexion (*p* = 0.001, η^2^ = 0.253) and extension kinaesthesia (*p* = 0.002, η^2^ = 0.212), 30° (*p* < 0.001, η^2^ = 0.408) and 60° position senses (*p* = 0.033, η^2^ = 0.109), AP (*p* < 0.001, η^2^ = 0.611) and ML displacement (*p* < 0.001, η^2^ = 0.849), and AP (*p* < 0.001, η^2^ = 0.558) and ML speed (*p* < 0.001, η^2^ = 0.502) decreased by 36.2%, 32.3%, 40.0%, 18.9%, 30.2%, 44.2%, 38.4%, and 24.0%, respectively, indicating a statistically significant difference. In the control group, the knee extension (*p* = 0.004, η^2^ = 0.201) with PT (60°/s) increased by 18.8%, showing a statistically significant difference. The AP (*p* < 0.001, η^2^ = 0.332) and ML displacement (*p* < 0.001, η^2^ = 0.779) and the AP (*p* < 0.001, η^2^ = 0.565) and ML speed (*p* = 0.002, η^2^ = 0.221) decreased by 14.9%, 40.0%, 26.8%, and 19.5%, respectively, showing a statistically significant difference.

A comparison between the groups was performed after 4 weeks. The experimental group exhibited the following improvements compared with the control group: the knee flexion (*p* = 0.011, η^2^ = 0.081) and extension (*p* = 0.014, η^2^ = 0.076) with PT (60°/s), extension (*p* = 0.026, η^2^ = 0.062) with PT (240°/s), and extension PT (*p* = 0.036, η^2^ = 0.055) increased, and the differences were statistically significant. However, the knee extension kinaesthesia (*p* = 0.048, η^2^ = 0.049), 30° position sense (*p* = 0.015, η^2^ = 0.074), and AP displacement (*p* = 0.001, η^2^ = 0.123) significantly decreased.

## Discussion

This study explored the effects of regular isokinetic muscle strength training on the injured knee joint, muscle strength, proprioception, and balance ability of athletes with ACL reconstruction. We tested whether adding regular isokinetic muscle strength training to rehabilitation training can further improve the knee flexion, extensor strength, and extensor endurance in athletes with ACL reconstruction and improve the balance of kinaesthesia, 30° position sense, and front and rear directions compared with aerodynamic resistance training. However, the proposed treatment had limited effects on knee flexion kinaesthesia and muscle endurance.

### Muscle strength

After 4 weeks, the knee flexion and extension torque (60°/s and 240°/s) and the flexion and extension muscular endurance increased by 31.7%, 40.3%, 23.4%, 42.9%, 21.4%, and 19.7%, and the control knee extension torque (60°/s) increased by 18.8%. Compared with the control group, the knee flexion and extension torque (60°/s), extension torque (240°/s), and extension muscular endurance increased to varying degrees. These results showed that isokinetic muscle strength training could improve the knee flexion, extensor strength, and extensor endurance of athletes with ACL reconstruction more than aerodynamic resistance training. Insufficient knee flexion and extensor strength in athletes with ACL reconstruction increases the risk of sports injury, such as ligament tear or joint pain (Buckthorpe et al., 2019). Thus, knee flexion and extensor group strength recovery is important in rehabilitation. Knee muscle strength was tested in 17 football players with ACL reconstruction, and the results showed that rapid strength recovery is important to return to play (Jacopetti et al., 2016). In another study, 15 athletes with ACL reconstruction were subjected to 24 weeks of isokinetic muscle strength training, and the results showed that their extensor muscle had a 60°/s peak moment, and the elevation was greater than that of the flexor muscle (Zhang et al., 2016). This study further validated and expanded the results of previous studies (Jacopetti et al., 2016; Zhang et al., 2016), which reported that the knee flexion and extension torque (60°/s and 240°/s) exceeded that of the flexor muscles in both the experimental and control groups. No scholar has reported on the change in the muscle endurance of athletes with ACL reconstruction. Thus, this study used the isokinetic muscle strength test system for analyzing muscle endurance knee flexion and extension. Compared with the pneumatic training group (control group), rehabilitation with increased isokinetic muscle strength training improved knee extension muscle endurance but did not add to knee flexion muscle endurance. The analysis showed that the intervention time was insufficient, and the knee flexion muscle endurance change delayed the extensor muscle endurance. Athletes have high requirements for muscle endurance, so the training of muscle endurance cannot be ignored in the rehabilitation process of athletes with ACL reconstruction. Improving the muscle endurance level plays an important role in the recovery of the competitive ability of athletes with ACL reconstruction.

### Proprioception

After 4 weeks, the knee flexion and extension kinaesthesia decreased by 30°, 36.2%, 32.3%, 40.0%, and 18.9%. Compared with the control group, the kinaesthesia and 30° position sense of the knee extension decreased in the experimental group. These results showed that isokinetic muscle strength training could further promote the recovery of kinaesthesia and 30° position sense in athletes with ACL reconstruction. The knee proprioceptors are divided into the Pacini and Lafini bodies, which are distributed in the joint capsule, cruciate ligament, collateral ligament, meniscus, and tendon. They can feel the deformation and pressure of tissues and perceive the process of accelerated initiation and deceleration of joint movements (Cheng et al., 2017). Studies confirmed the presence of mechanical stimulation receptors in ACL as important proprioceptive carriers (Larwa et al., 2021; Kotsifaki et al., 2023). Consequently, ACL reconstruction impairs proprioception in athletes. No uniform method exists for proprioception training in patients with ACL reconstruction, and this study showed that isokinetic muscle strength training improved the proprioception of athletes after ACL reconstruction. A previous study (Jerosch and Prymka, 1996) explained that the 30° position sense leads to more significant changes than the 60° position sense, and kinaesthesia significantly improves compared with flexion. In addition, the 30° position sense may be related to the design of isokinetic muscle strength training, during which the subjects are required to recruit more muscle fibres to achieve the training effect at the beginning of the exercise. In this study, the peak muscle strength moment appeared at approximately 30°. The mechanism by which isokinetic muscle strength training improves subjects’ proprioception was analysed through neuromuscular strengthening and promotion of cognitive level processes (Khademi et al., 2022). In addition, isokinetic muscle strength training is a periodic and stable exercise (Cheng and Jiang, 2020), which can release the elastic energy stored in the subject’s tendon structure, strengthen the muscle tactile reflex, and promote the recovery of proprioception by improving the central nervous system.

### Balance ability

After 4 weeks, the experimental group’s displacement and speed of the knee joint decreased by 30.2%, 44.2%, 38.4%, and 24.0%. The control group’s AP and ML displacement and speed decreased by 14.9%, 40.0%, 26.8%, and 19.5%. AP displacement was reduced in the experimental group compared with that in the control group, indicating that isokinetic muscle strength training further promoted the recovery of balance ability in athletes with ACL reconstruction. Arockiaraj et al. (2013) believed that the altered signal afferents of the injured limb of the ACL will affect the muscle spindle function of the healthy limb and cause impairment in the healthy limb’s proprioception function, thereby affecting the balance ability of the human body. Previous studies have shown that patients with ACL reconstruction have the highest fall risk at 4 weeks, which gradually decreases at 8–12 weeks after surgery (Gokalp et al., 2016). The present study found that isokinetic muscle strength training further improved the AP direction balance compared with changes in the left–right direction balance. This finding may be attributed to the knee flexor and extensor muscle group exercise. Given that the left and right directions of the human body do not often exercise, no additional improvement effect in the left and right directions may be observed. This study examined regular isokinetic muscle strength training in athletes after ACL reconstruction for changes in maintaining human postural control (knee muscle strength, proprioception, and balance). The results provided a quantitative basis for scientific rehabilitation training. Athletes with ACL reconstruction should add regular isokinetic muscle strength training to rehabilitation training, which is an effective rehabilitation strategy.

This study had some limitations, including limited samples to explore gender differences and insufficient neuromuscular response and gait testing. The influence of distinct exercise habits amongst athletes from different sports disciplines must be eliminated. Athletes from the same sports discipline were not selected as a group. In addition, we did not use long intervention times and long-term follow-up evaluations, which will be addressed in future work.

## Conclusion

This study showed that adding regular isokinetic muscle strength training to rehabilitation training could improve the knee flexion, extensor strength, extensor endurance, kinaesthesia, 30° position sense, and balance compared with aerodynamic resistance training. However, insignificant effects on knee flexion kinaesthesia and muscular endurance were observed.

## Data Availability

The raw data supporting the conclusion of this article will be made available by the authors, without undue reservation.
